# Efficacy and safety of 3‐n‐butylphthalide for the treatment of cognitive impairment: A systematic review and meta‐analysis

**DOI:** 10.1111/cns.13952

**Published:** 2022-09-01

**Authors:** Qiulu Zhou, Chao Han, Yun Xia, Fang Wan, Sijia Yin, Yunna Li, Liang Kou, Xiaosa Chi, Junjie Hu, Yadi Sun, Jiawei Wu, Wenkai Zou, Jinsha Huang, Tao Wang

**Affiliations:** ^1^ Department of Neurology Union Hospital, Tongji Medical College, Huazhong University of Science and Technology Wuhan Hubei China; ^2^ Department of Neurology The First Affiliated Hospital of USTC, Division of Life Sciences and Medicine, University of Science and Technology of China Hefei Anhui China

**Keywords:** 3‐n‐butylphthalide, cognitive impairment, meta‐analysis, MMSE, MoCA

## Abstract

**Background:**

Current evidence for the efficacy of pharmacological treatment in improving cognitive function is absent. Recent studies have reported that 3‐n‐butylphthalide (NBP) has a positive effect on improving cognitive impairment; however, its clinical efficacy and safety is unclear. Therefore, we conducted a meta‐analysis to assess its efficacy and safety for cognitive impairment.

**Methods:**

We systematically searched the PubMed, EMBASE, Cochrane Library, Web of Science, and Scopus databases, and two reviewers independently screened and extracted the data from included studies. We synthesized the data using the Review Manager Software version 5.3.

**Results:**

We included six randomized clinical trials (RCTs), encompassing 851 patients with cognitive impairment. The results showed that NBP improved cognitive impairment. Specifically, the clinical efficacy was better than that in the control group, with better performance in improving the Mini‐Mental State Examination and the Montreal Cognitive Assessment scores, while decreasing the Alzheimer's Disease Assessment Scale‐Cognitive subscale and the Clinician's Interview‐Based Impression of Change plus caregiver input scores. There was no significant difference in the incidence of adverse events between both groups.

**Conclusion:**

The NBP is effective and safe in improving cognitive impairment; however, more high‐quality RCTs are needed to confirm these findings.

## Graphical abstract

Objectives: To evaluate the efficacy and safety of NBP in the treatment of patients with cognitive impairment. Results: Six RCTs encompassing 851 patients with cognitive impairment were included. The results indicated that NBP had better performance in improving the cognitive impairment compared to the control group. There was no significant difference in the incidence of adverse events between the two groups. Conclusion: NBP could improve cognitive impairment caused by cerebrovascular diseases and Parkinson's disease and is rather safe.

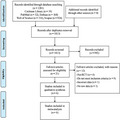

## 1 INTRODUCTION

As the population ages, the number of people with cognitive impairment is increasing.^1^ Many diseases cause cognitive impairment, including cerebrovascular diseases,^2^ neurodegenerative diseases,^3^ and acute carbon monoxide poisoning‐related encephalopathy. Types of cognitive impairment include mild cognitive impairment (MCI) and dementia.^4^ In 2015, the number of people with dementia was 47 million worldwide, and by 2050, this number will triple.^5^ This will place huge disease and financial burdens on individuals, families, and public health services.^6^ Therefore, effective treatments for cognitive impairment are of great social and clinical significance.

Currently, drugs commonly used to improve cognitive impairment include cholinesterase inhibitors (donepezil, rivastigmine, and galantamine) and the N‐methyl‐D‐aspartate (NMDA) receptor antagonist memantine. However, none of these drugs can halt disease progression, and their therapeutic efficacy for MCI and dementia remains controversial.^7^, ^8^ In addition, their use in the conventional treatment of cognitive impairment is limited because of the numerous side effects and unclear pathological mechanisms. The mechanisms underlying cognitive impairment have not been fully elucidated. Several studies have indicated that the main pathogenesis might be related to disturbances in energy metabolism, oxidative damage, mitochondrial dysfunction, and neuronal death.^9^, ^10^ Therefore, new effective drugs with neuroprotective effects are needed to treat cognitive impairment, especially multitargeted drugs with ameliorated pathogenesis.

3‐n‐Butylphthalide (NBP), a pure component extracted from Apium graveolens Linn, was approved by the China Food and Drug Administration in 2002 for the treatment of ischemic stroke because of its protective effects against cerebral ischemia.^11^ The mechanism might be that NBP inhibits neuronal apoptosis by modulating the Akt/mTOR^12^ and GDNF/GFRAK1/Ret^13^ signaling pathways. Notably, a recent randomized multicenter clinical trial has shown that NBP exerts a protective effect on vascular cognitive impairment.^14^ The underlying mechanism may be related to the multitargeted protective effects of NBP, such as the reduction of oxidative damage^15^ and inflammatory response,^16^ improvement of mitochondrial function, and inhibition of apoptosis.^17^, ^18^ Moreover, another clinical trial has shown that NBP exerts beneficial effects on cognitive impairment induced by brain microcirculatory disorders and mitochondrial dysfunction in Alzheimer's disease (AD).^19^ More than 80% of patients with Parkinson's disease (PD) frequently develop cognitive impairment 5 years after diagnosis, and considering that the number of PD patients is expected to exceed 12 million by 2040,^20^ this will place a heavy social and economic burden on individuals, families, communities, and countries.^21^, ^22^ We previously showed that NBP improved PD motor symptoms in a mouse model.^23^ In addition, NBP can alleviate cognitive symptoms by modulating mitochondrial dynamics.^24^ Therefore, NBP might be an emerging drug for improving cognitive impairment.

In recent years, NBP has been increasingly used to treat cognitive impairment; however, the number and sample sizes of completed clinical studies of NBP for cognitive impairment are relatively small. Thus, the results of these studies are not concrete to provide guidance for its clinical application in cognitive impairment. Hence, it is necessary to reassess its clinical efficacy and safety. This study aimed to analyze and evaluate the efficacy and safety of NBP in cognitive impairment, provide a new reference for the clinical treatment of cognitive impairment, and promote the development in the field of cognitive impairment.

## 2 METHODS

### 2.1 Search strategies

We used the Preferred Reporting Items for Systematic Reviews and Meta‐Analyses statement to guide the reports in this systematic review and meta‐analysis.^25^ PubMed, EMBASE, Cochrane Library, Web of Science, and Scopus databases were systematically searched by two reviewers (Xia and Wan) independently from inception to February 23, 2022. Using a combination of medical subject headings and free‐text terms, with PubMed as an example, the specific search strategy is shown in Table S1.

### 2.2 Inclusion criteria

Two reviewers (Zhou and Han) independently screened and examined all the identified studies in accordance with the principle: population, interventions, comparisons, outcomes, and study design. Disagreements were resolved by consensus between the two reviewers or by the corresponding author (Wang). The inclusion criteria were as follows: (1) population: patients with a diagnosis (as determined by the studies' authors) of cognitive impairment or dementia were chosen regardless of disease type, race, sex, or age; (2) interventions: the treatment group received NBP alone or in combination with conventional Western medicine (CWM) treatment, while the control group received placebo, the same CWM, or routine treatment. There were no restrictions on the dose, dosage form, or mode of administration for NBP. In addition, there were no restrictions on the duration of treatment; however, the two groups had to be the same throughout the study. The CWM must be recognized as a definite drug to improve cognitive impairment, such as memantine, donepezil, and piracetam.^26^, ^27^ (3) Outcomes: clinical efficacy (depending on whether the patient's clinical symptoms or Mini‐Mental State Examination [MMSE] scores improve, the details are determined by the authors of the included studies). Global cognitive function can be clinically measured using internationally recognized assessment scales, such as the MMSE, Montreal Cognitive Assessment (MoCA), Alzheimer's Disease Assessment Scale‐Cognitive subscale (ADAS‐cog), and the Clinician's Interview‐Based Impression of Change plus caregiver input (CIBIC‐plus). We assessed the clinical safety of NBP by analyzing the occurrence of adverse events or adverse reactions. (4) Study design: we included randomized controlled trials (RCTs).

### 2.3 Exclusion criteria

Studies that met one of the following criteria were excluded: (1) not RCTs (animal‐related studies, case reports, reviews, letters, or editorials); (2) studies not published in English; (3) studies not available in full text (after trying various ways and corresponding with the authors); (4) studies with uncertain outcome data; (5) studies with unavailable cognitive impairment outcomes; and (6) irrelevant studies.

### 2.4 Data extraction

Two reviewers (Zhou and Han) independently evaluated and extracted data from all candidate studies. Discrepancies were resolved by consensus or with assistance from the corresponding author (Wang). The data extracted from the included studies consisted of the following: general characteristics of the trial (first author, year of publication), mean age, total number of participants and males, disease course, intervention, duration of therapy, type of disease, MMSE or MoCA baseline scores, and outcomes. When the outcomes were evaluated at different follow‐up times, those with the longest follow‐up time were selected.

### 2.5 Quality assessment

Two reviewers (Zhou and Han) independently assessed the quality of the included studies using Cochrane Collaboration's risk of bias tool. If there was any disagreement, a consensus was reached through negotiation or consultation with the corresponding author (Wang). The Cochrane Collaboration's risk of bias tool consists of seven items and each item's risk of bias is classified as high, unclear, or low.^28^

### 2.6 Subgroup analysis

Considering the different disease types included in the eligible studies, subgroup analysis was performed according to the following types: vascular cognitive impairment without dementia, vascular dementia (VD), and Parkinson's disease with dementia (PDD). Regardless of whether NBP is a monotherapy or a combination therapy.

### 2.7 Statistical analysis

We used the Review Manager software (version 5.3) for meta‐analysis. We presented continuous data as standardized mean differences (SMD) and 95% confidence intervals (CI), and displayed dichotomous data as risk ratios (RR) and 95% CI. We used the I‐squared statistic (*I* ^2^) and Q statistic to evaluate the heterogeneity of the eligible studies. The *I* ^2^ > 50% and *p* < 0.1 suggested significant heterogeneity among the results. It was then assessed using the Mantel–Haenszel random‐effects model; otherwise, the fixed‐effects model was employed to pool the data if the heterogeneity was acceptable (*I* ^2^ ≤ 50%, *p* ≥ 0.1). We used the Z‐test to evaluate the significance of the pooled results, with *p* < 0.05 designated as a statistically significant difference.

For a three‐arm or multiarm test, multiple subgroups of data can be combined, and the following formula was used to combine continuous data (assuming the number of subgroups was two, the sample sizes of the two subgroups were *N*_1_ and *N*_2,_ respectively; the means were *M*_1_ and *M*_2,_ respectively; the standard deviations (SDs) were SD_1_ and SD_2,_ respectively; the overall mean was Mean_T_; and the overall standard deviation was SD_T_)^28^:

$${\text{Mean}}_{T}=\frac{N_{1}M_{1}+N_{2}M_{2}}{N_{1}+N_{2}}$$

$${\mathrm{SD}}_{T}=\sqrt{\frac{\left(N_{1}-1\right){\mathrm{SD}}_{1}^{2}+\left(N_{2}-1\right){\mathrm{SD}}_{2}^{2}+\frac{N_{1}N_{2}}{N_{1}+N_{2}}\left(M_{1}^{2}+M_{2}^{2}-2M_{1}M_{2}\;\right)}{N_{1}+N_{2}-1}}$$

In some trials, the mean and SD, which changed from baseline to each evaluation time, were not reported. Therefore, we calculated SD using the SDs of the baseline and the final evaluation time.^28^, ^29^ The calculated results are shown in Supplementary data.

## 3 RESULTS

### 3.1 Study inclusion

**FIGURE 1:**
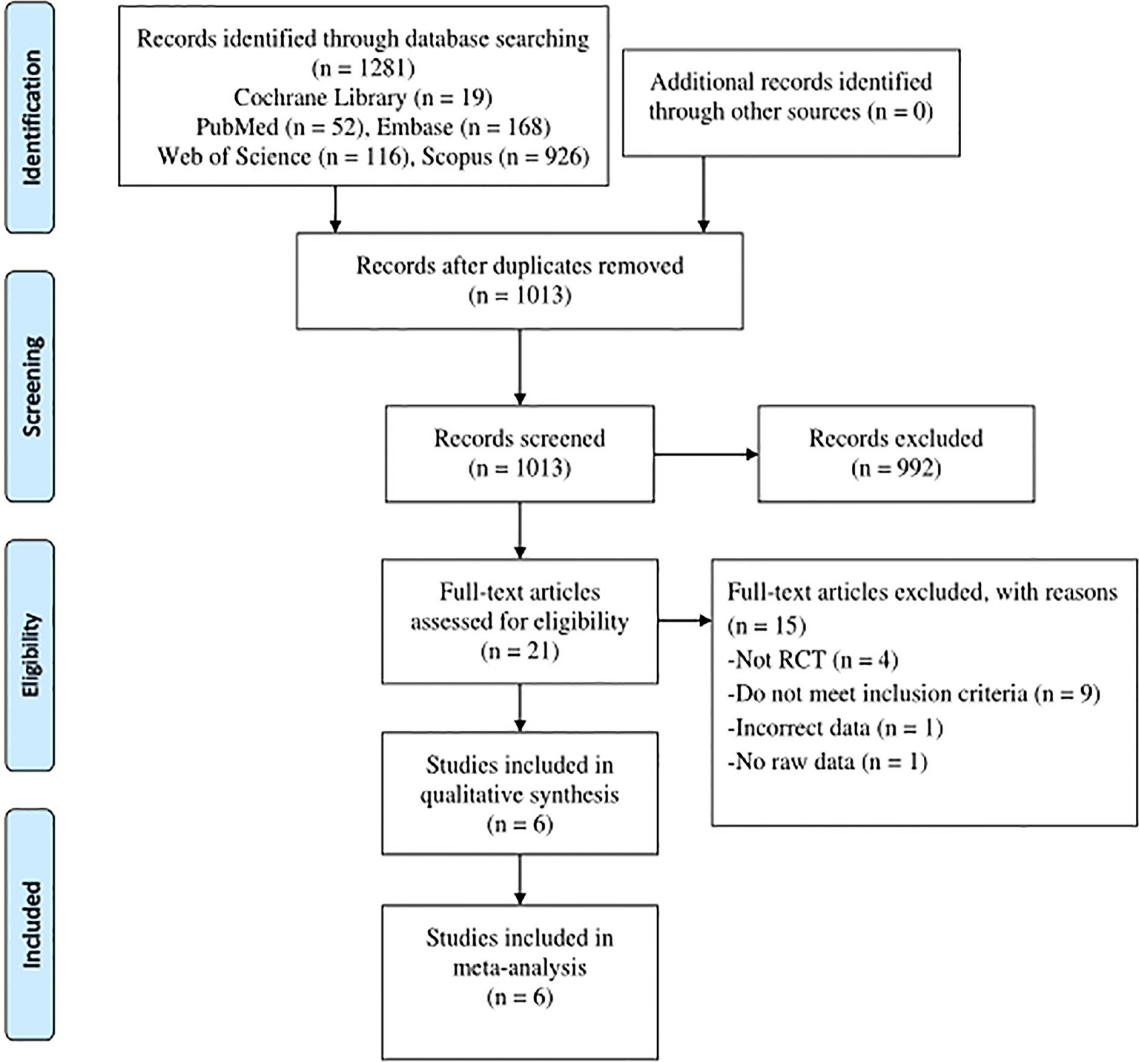
Literature screening flow chart

Initially, we examined 1281 studies. After removing 268 duplicates, we reviewed the titles and abstracts of the studies and excluded 992. Four relevant ongoing studies were found (Table 1). Of the 21 remaining studies, 15 were excluded because they did not meet the inclusion criteria, raw data were not available, and incorrect data (Table 2). Finally, six studies^14^, ^30^, ^31^, ^32^, ^33^, ^34^ comprising 851 patients were included, of which 446 patients were in the treatment group. The screening process is presented as a flow chart in the literature (Figure 1).

**TABLE 1:** Characteristics of ongoing studies

Main ID	ChiCTR1800018362^54^	ChiCTR1800017084^55^	NCT04078204/2018ZX09711001‐003‐021^56^	ChiCTR2000032555^57^
Scientific title	Efficacy and safety of butylphthalide on patients with mild cognitive impairment: a multicenter, randomized, double‐blind, placebo‐controlled trial	The efficacy of butylphthalide on cognitive impairment of patients with idiopathic PD and DBS patients: A randomized, double‐blind, placebo‐controlled, multicenter trial	A multicenter, randomized, double‐Blind, placebo‐controlled study of dl‐3‐butylphthalide on the treatment of small cerebral vessel disease	A 24‐week multicenter, randomized controlled study to evaluate the efficacy of NBP on delayed‐onset post stroke cognitive impairment
Methods	RCT	RCT	RCT	RCT
Participants	270 participants	280 participants	300 participants	3000 participants
Interventions	NBP versus Placebo	NBP versus Placebo	NBP versus placebo	NBP and Routine treatment versus Routine treatment
Outcomes	ADAS‐Cog; MMSE; CDR; NPI; ADL; Immediate memory; Delayed recall; Digit span; Trail making test; FDG‐PET	MMSE; MoCA; MDS‐UPDRS part III, IV; SCOPA‐Cog; PDQ‐39; CGI‐I; HAMD; PDSS	CIBIC‐Plus; ADAS‐cog; mRS; MMSE; CDR‐SB; IADLs	Magnetic resonance imaging; Cognitive and psychosomatic correlation scales; Plasma inflammatory factor
Contact information	Cuibai Wei and Tingting Li, Xuanwu hospital, Capital Medical University, 45 Changchun Street, Xicheng District, Beijing, China	Junjie Hu and Tao Wang, Union Hospital, Tongji Medical College, Huazhong University of Science and Technology, 13 Hangkong Road, Qiaokou District, Wuhan, Hubei, China	Ruixin Pan, Peking Union Medical College Hospital, Beijing, China	Wenhui Lu and Qiumin Qu, The First Affiliated Hospital of Xi'an Jiaotong University, 277 Yanta Road West, Yanta District, Xi'an, Shaanxi, China
Date of first enrolment	October, 2018	August, 2018	April, 2018	April, 2020

Abbreviations: ADAS‐Cog, Alzheimer's Disease Assessment Scale‐Cognitive subscale; ADL, Activities of Daily Living; CDR, Clinical Dementia Rating scale; CDR‐SB, Clinical Dementia Rating Scale Sum of Boxes; CIBIC‐Plus, Clinician's Interview‐Based Impression of Change Plus caregiver input; DBS, Deep Brain Stimulation; FDG‐PET, Fluorodeoxyglucose Positron Emission Tomography; HAMD, Hamilton Depression scale; IADLs, Instrumental Activities of Daily Living; MDS‐UPDRS part III, IV, Movement Disorder Society‐Sponsored Revision of the Unified Parkinson's Disease Rating Scale part III, IV; MMSE, Mini‐Mental State Examination; MoCA, Montreal Cognitive Assessment; mRS, modified Rankin Score; NBP, 3‐n‐Butylphthalide; NPI, Neuropsychiatric Inventory; PD, Parkinson's Disease; PDQ‐39, Parkinson's Disease Questionnaire‐39; CGI‐I, Clinical Global Impression‐Improvement scale; PDSS, Parkinson's Disease Sleep Scale; RCT, Randomized Controlled Trial; SCOPA‐Cog, Scales for Outcomes in Parkinson's Disease‐Cognition.

**TABLE 2:** Reasons of excluded studies

Study	Reasons for exclusion
Zhang 2014,^58^ Yan 2017,^59^ Zhang 2020,^60^ Wang 2021^19^	Not an RCT
Lu 2016,^61^ Zhan 2017,^62^ Fan 2018,^63^ Wu 2018,^64^ Yuan 2018,^65^ Zhang 2018,^66^ Sun 2020,^67^ Wu 2020,^68^ Xu 2021^69^	No meeting inclusion criteria
Qi 2020^70^	Incorrect data
Xiang 2017^71^	No raw data

Abbreviation: RCT, Randomized Controlled Trial.

### 3.2 Characteristics of included studies

This meta‐analysis finally included six RCTs.^14^, ^30^, ^31^, ^32^, ^33^, ^34^ All included studies were published in English between 2016 and 2020, with sample sizes ranging from 60 to 280 patients. The disease types in the studies were VD, PDD, and vascular cognitive impairment without dementia. All six studies used the internationally recognized scales to evaluate global cognitive function: four studies^14^, ^30^, ^32^, ^33^ used the MMSE, three studies^31^, ^32^, ^34^ used the MoCA, and one study^14^ used the ADAS‐cog and the CIBIC‐plus. In addition, different comparisons were employed, including NBP versus routine treatment, NBP versus placebo, NBP versus CWM, and NBP plus CWM versus CWM. The duration of therapy ranged from three to six months, and adverse events were reported in all the studies. The detailed characteristics of each study are summarized in Table 3.

**TABLE 3:** The characteristics of all included studies

Study	Mean age, year (Mean ± SD)	*N* Total number of participants (*N* Male)	Disease course (Mean ± SD)	Intervention (drug dosage)	Duration of Therapy, month	Type of disease	MMSE or MoCA baseline scores (Mean ± SD)	Outcomes
Jia et al^14^	T: 68.00 ± 8.80 C: 66.70 ± 7.70	T: 140 (92) C: 140 (92)	T: NR C: NR	T: NBP (200 mg, Tid) C: Placebo (200 mg, Tid)	6	Vascular cognitive impairment without dementia	T: MMSE: 25.01 ± 2.49 C: MMSE: 25.10 ± 2.37	MMSE; ADAS‐cog; CIBIC‐plus; adverse events
Chen at al^30^	T: 63.42 ± 4.88 C: 62.58 ± 4.73	T: 86 (43) C: 86 (44)	T: 16.14 ± 4.72 (months) C: 15.36 ± 4.08 (months)	T: NBP (200 mg, Tid) + Routine treatment C: Piracetam (800 mg, Tid) + Routine treatment	3	VD	T: MMSE: 12.64 ± 2.80 C: MMSE: 12.37 ± 2.61	clinical efficacy; MMSE; adverse events
Ma et al^31^	T: 64.30 ± 3.95 C: 64.28 ± 3.94	T: 82 (52) C: 41 (29)	T: 2.61 ± 0.49 (years) C: 2.70 ± 0.61 (years)	T: NBP (200 mg, Tid) + Routine treatment C: Routine treatment	3	VD	T: MoCA: 18.17 ± 2.7 C: MoCA: 18.76 ± 2.95	clinical efficacy; MoCA; adverse events
Peng et al^32^	T: 68.50 ± 4.30 C: 68.90 ± 4.60	T: 46 (26) C:46 (25)	T: 7.92 ± 2.21 (years) C: 7.69 ± 2.18 (years)	T: NBP (200 mg, Bid) + Donepezil (one tablet, Qd) + Routine treatment C: Donepezil (one tablet, Qd) + Routine treatment	4	PDD	T: MMSE: 21.36 ± 3.38 C: MMSE: 22.15 ± 3.12 T: MoCA: 18.26 ± 4.27 C: MoCA: 18.79 ± 4.58	clinical efficacy; MMSE; MoCA; adverse events
Wang et al^33^	T: 66.75 ± 5.78 C: 66.18 ± 6.35	T: 62 (38) C: 62 (35)	T: 17.23 ± 6.25 (months) C: 17.65 ± 5.94 (months)	T: NBP (200 mg, Tid) + Routine treatment C: Donepezil (5 mg, Qd) + Routine treatment	3	VD	T: MMSE: 18.17 ± 4.25 C: MMSE: 18.63 ± 4.88	MMSE; adverse events
Zhang et al^34^	T: 68.80 ± 8.70 C: 67.90 ± 9.50	T: 30 (16) C: 30 (16)	T: 1.90 ± 0.90 (years) C: 1.80 ± 0.80 (years)	T: NBP (200 mg, Tid) + Donepezil (10 mg, Qd) + Oxiracetam (800 g, Tid) + Ginkgo biloba extract (80 mg, Tid) + Routine treatment C: Donepezil, (10 mg, Qd) + Routine treatment	6	PDD	T: MoCA: 16.19 ± 2.14 C: MoCA: 16.37 ± 2.61	MoCA; adverse events

Abbreviations: ADAS‐Cog, Alzheimer's Disease Assessment Scale‐Cognitive subscale; C, Control; CIBIC‐Plus, Clinician's Interview‐Based Impression of Change Plus caregiver input; MMSE: Mini‐Mental State Examination; MoCA, Montreal Cognitive Assessment; NBP, 3‐n‐Butylphthalide; NR, Not reported; PPD, Parkinson's Disease with Dementia; T, Trail; VD, Vascular Dementia.

### 3.3 Methodological quality assessment

**FIGURE 2:**
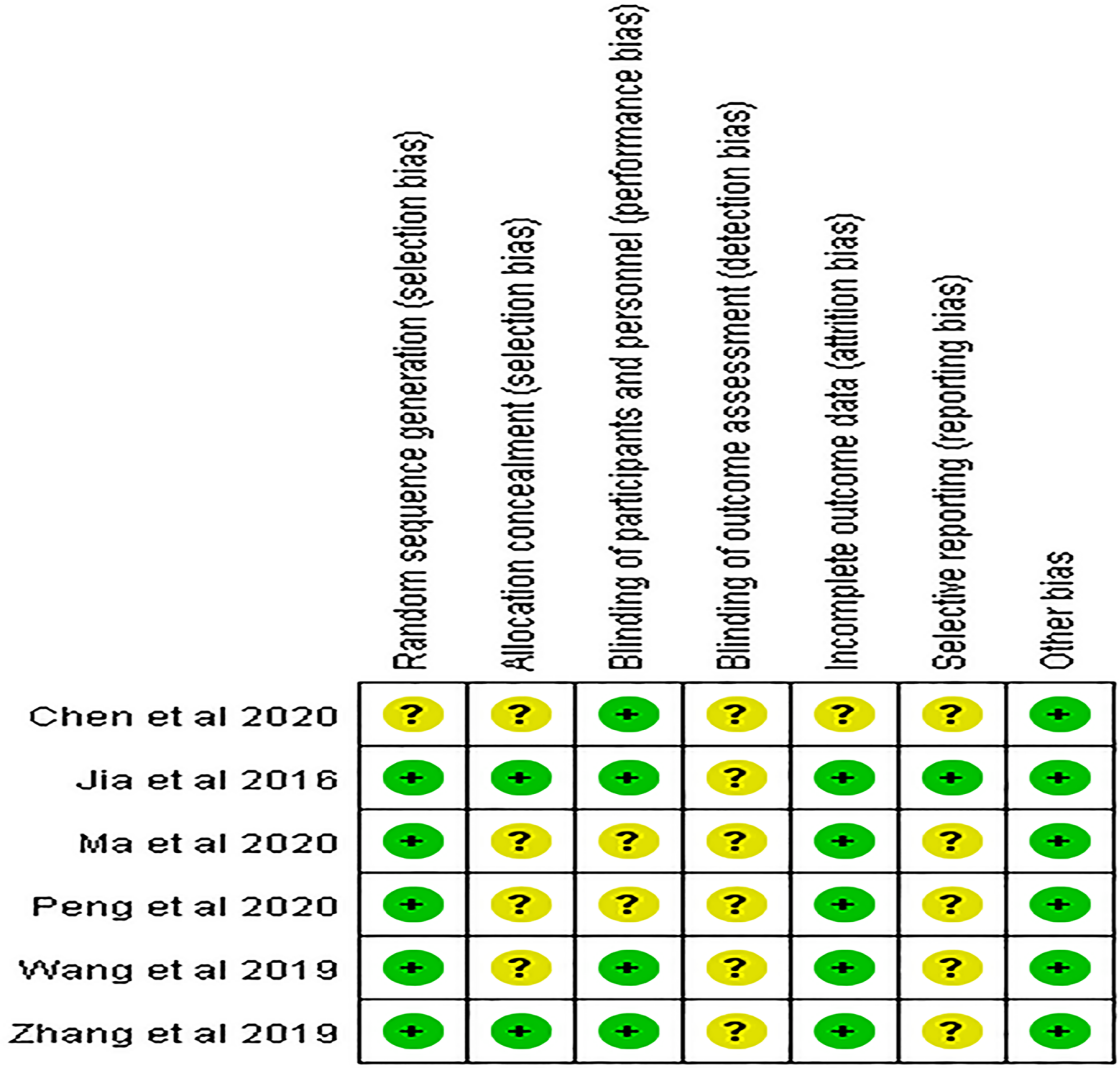
Risk of bias summary of included studies

The Cochrane Collaboration's risk of bias tool was used to evaluate the methodological quality of each study. Five RCTs^14^, ^31^, ^32^, ^33^, ^34^ reported on their method of generating sequences using random number tables or SAS software, while a study^30^ was rated as “unclear risk” because it only mentioned “randomization” and did not provide further details. Only two studies^14^, ^34^ used sealed envelopes or kits to conceal allocations. Four studies^14^, ^30^, ^33^, ^34^ clarified the blinding of the participants and personnel. All studies^14^, ^30^, ^31^, ^32^, ^33^, ^34^ were rated as “unclear risk” because they did not elaborate on the blinding of outcome assessors. Five studies^14^, ^31^, ^32^, ^33^, ^34^ were rated as “low risk” and one of these^14^ applied an appropriate intention‐to‐treat analysis. The reporting bias of five RCTs^30^, ^31^, ^32^, ^33^, ^34^ was rated as “unclear risk” due to a lack of registration number. No other bias was found in the included studies (Figure 2).

### 3.4 Outcomes of NBP for cognitive impairment

#### 3.4.1 Clinical efficacy

**FIGURE 3:**
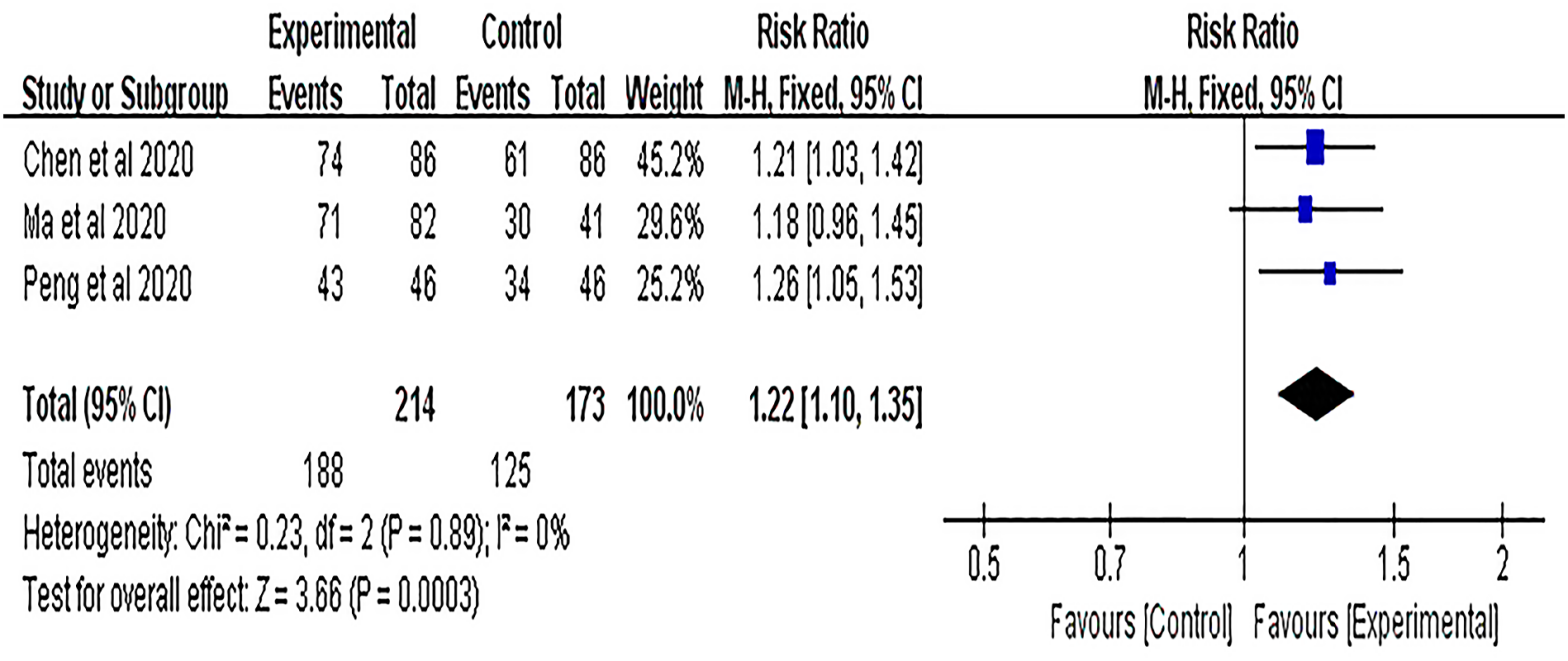
Meta‐analysis results of clinical efficacy

Clinical efficacy was mentioned in three studies,^30^, ^31^, ^32^ including 387 patients. No obvious heterogeneity was observed in these studies (*I* ^2^ = 0%, *p* = 0.89); therefore, a fixed‐effects model was used for the analysis. The pooled results showed that the clinical efficacy of the NBP group was better than that of the control group, and the difference was statistically significant (RR = 1.22, 95% CI = [1.10, 1.35], *p* = 0.0003; Figure 3).

#### 3.4.2 Global cognitive function

##### MMSE

**FIGURE 4:**
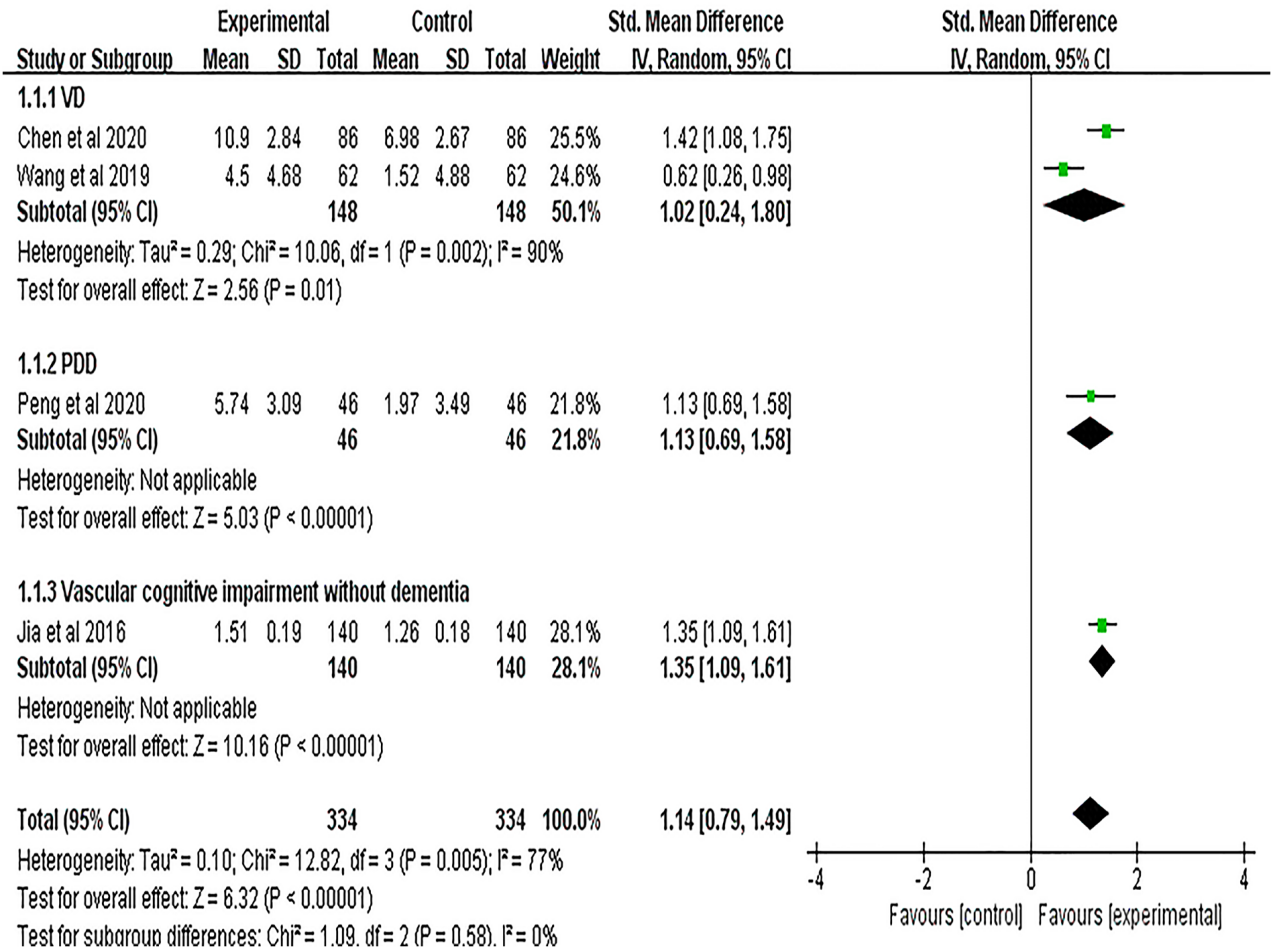
Meta‐analysis results for the effect of NBP treatment on MMSE. VD, Vascular Dementia; PDD, Parkinson's Disease with Dementia

Four studies^14^, ^30^, ^32^, ^33^ used the MMSE to assess cognitive function, and significant heterogeneity was observed (*I* ^2^ = 77%, *p* = 0.005). The subgroup analysis of different disease types showed that two studies^30^, ^33^ had VD, showing significant heterogeneity (*I* ^2^ = 90%, *p* = 0.002), while one study^32^ had PDD and the other^14^ had vascular cognitive impairment without dementia, both with heterogeneity not applicable. Pooled results using the random‐effects model showed that the NBP group had significantly improved scores on the MMSE (VD: SMD = 1.02, 95% CI = [0.24, 1.80], *p* = 0.01; PDD: SMD = 1.13, 95% CI = [0.69, 1.58], *p* < 0.00001; vascular cognitive impairment without dementia: SMD = 1.35, 95% CI = [1.09, 1.61], *p* < 0.00001; Figure 4).

##### MoCA

**FIGURE 5:**
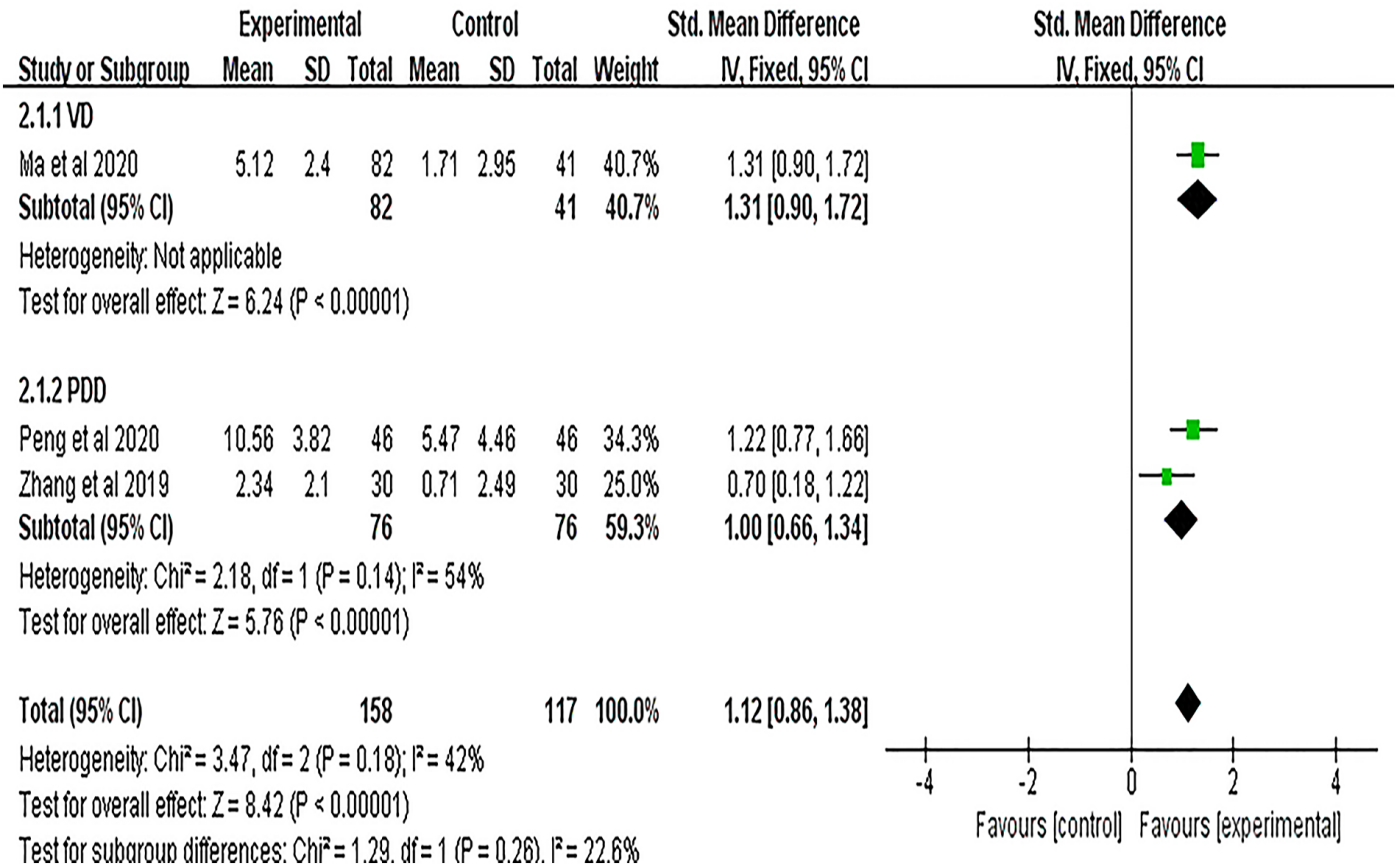
Meta‐analysis results for the effect of NBP treatment on MoCA. VD, Vascular Dementia; PDD, Parkinson's Disease with Dementia

Three studies^31^, ^32^, ^34^ used the MoCA to assess cognitive function and accepted heterogeneity was observed (*I* ^2^ = 42%, *p* = 0.18). The subgroup analysis of different disease types showed that one study^31^ had VD with inapplicable heterogeneity, and others had PDD,^32^, ^34^ showing acceptable heterogeneity (*I* ^2^ = 54%, *p* = 0.14). Pooled results using the fixed‐effects model showed that the NBP group performed better in improving MoCA scores in patients with cognitive impairment than the control group (VD: SMD = 1.31, 95% CI = [0.90, 1.72], *p* < 0.00001; PDD: SMD = 1.00, 95% CI = [0.66, 1.34], *p* < 0.00001; Figure 5).

##### ADAS‐cog and CIBIC‐plus

A study^14^ evaluated changes in ADAS‐cog and CIBIC‐plus scores after NBP treatment. Compared to the control group, the NBP group showed better performance in decreasing ADAS‐cog scores (SMD = −3.05, 95% CI = (−3.39, −2.70), *p* < 0.00001). The changes in the CIBIC‐plus score were similar (SMD = −4.13, 95% CI = (−4.55, −3.71), *p* < 0.00001).

#### 3.4.3 Adverse events

**FIGURE 6:**
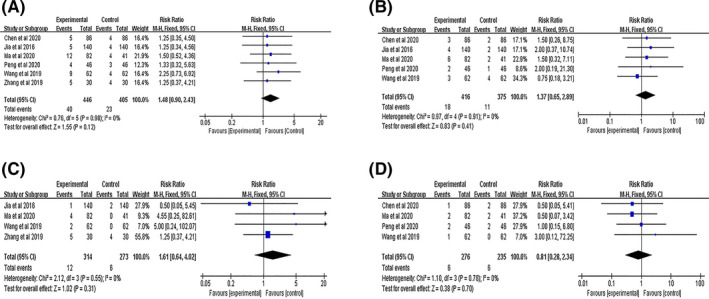
Meta‐analysis results of (A) total adverse events, (B) gastrointestinal side effects, (C) abnormal liver function, (D) neurological side effects

All six studies^14^, ^30^, ^31^, ^32^, ^33^, ^34^ reported adverse events. There was no significant heterogeneity (*I* ^2^ = 0%, *p* = 0.98) in these studies; therefore, a fixed‐effects model was used for analysis. The meta‐analysis showed no statistically significant difference between the two groups (RR = 1.48, 95% CI [0.90, 2.43], *p* = 0.12; Figure 6A). Of these, five studies^14^, ^30^, ^31^, ^32^, ^33^ reported gastrointestinal side effects (RR = 1.37, 95% CI [0.65, 2.89], *p* = 0.41; Figure 6B), including nausea, vomiting, and gastrointestinal discomfort. Four studies^14^, ^31^, ^33^, ^34^ reported abnormal liver function (RR = 1.61, 95% CI [0.64, 4.02], p = 0.31; Figure 6C). In addition, four studies^30^, ^31^, ^32^, ^33^ reported neurological side effects (RR = 0.81, 95% CI [0.28, 2.34], *p* = 0.70; Figure 6D), including insomnia, dizziness, fatigue, and psychiatric symptoms. Gastrointestinal side effects, abnormal liver function, and neurological side effects were common adverse events, and no serious adverse event was observed.

## 4 DISSCUSSION

In this systematic review, we evaluated the efficacy and safety of NBP for the treatment of cognitive impairment. Six published RCTs involving 851 patients were included in the meta‐analysis. The pooled data suggested that NBP was beneficial for improving clinical efficacy and global cognitive function measured by MMSE, MoCA, ADAS‐cog, and CIBIC‐plus. Furthermore, the results of the subgroup analysis showed that NBP improved different types of cognitive impairment such as vascular cognitive impairment without dementia, VD, and PDD. In addition, a recent meta‐analysis^35^ showed that NBP improved poststroke cognitive impairment (PSCI), a type of vascular cognitive impairment, which is generally consistent with the conclusions of our meta‐analysis. However, the meta‐analysis^35^ only focused on the improvement in PSCI with NBP. In contrast, our meta‐analysis showed that NBP could improve cognitive impairment resulting from multiple causes including PSCI. To some extent, our meta‐analysis might expand the application of NBP in the field of cognitive impairment and provide evidence for its wider application. Furthermore, there was no statistical difference in the incidence of adverse events (commonly gastrointestinal side effects, abnormal liver function, and neurological side effects) between the NBP and control groups. According to this meta‐analysis, the positive effects and safety of NBP on cognitive impairment, makes it a viable option in clinical practice.

The MMSE scale is the most commonly used measure for evaluating general cognitive function.^36^ In our review, four studies used MMSE to explore the therapeutic effects of NBP on cognitive impairment. Three types of diseases were included in the studies, namely, vascular cognitive impairment without dementia, VD, and PDD. Subgroup analysis was then performed and the results showed that NBP treatment improved all the types of cognitive impairment. In addition, we found that in the VD subgroup, two studies^30^, ^33^ compared the effects of NBP with CWM on cognitive impairment (Table 3), and the merged results indicated that NBP might be superior to CWM in improving MMSE scores. The MoCA scale is more specific than the MMSE scale in identifying mild cognitive impairment.^37^ Three studies used the MoCA scale. The disease type in two of these studies was PDD, and their treatments were both NBP plus CWM and CWM (Table 3). The pooled results showed that NBP plus CWM performed better than CWM alone on MoCA scores in patients with PDD. However, the small number of included studies limited our ability to assess the superiority of NBP alone or in combination for the treatment of cognitive impairment in different disease contexts. Future clinical trials of NBP for cognitive impairment could appropriately draw on the interventions and disease types described above.

Currently, cognitive impairment is primarily treated with pharmacological treatments, including cholinesterase inhibitors, donepezil, rivastigmine, and galantamine, in combination with the NMDA receptor antagonist, memantine.^38^ A meta‐analysis has shown that cholinesterase inhibitors and memantine produce minor cognitive benefits in patients with mild‐to‐moderate VD, with uncertain clinical significance.^39^ In addition, adverse events (mainly gastrointestinal side effects) are more frequent with cholinesterase inhibitors than with memantine.^39^ Several recent meta‐analyses^40^, ^41^ have shown that donepezil 5 mg/d, donepezil 10 mg/d, galantamine 16–24 mg/d, and memantine 20 mg/d slightly improve cognitive performance in patients with vascular cognitive impairment. There is evidence that 10 mg/d donepezil is more beneficial when adverse events are more.^41^ Therefore, based on the analysis of efficacy and safety acceptability, 20 mg/day memantine might be a better choice.^41^ Based on the above, data are not sufficient to support the widespread use of these medications in the treatment of vascular cognitive impairment. Similarly, cholinesterase inhibitors have certain effects on PDD. The currently available drug for PDD is rivastigmine, but it is associated with a high incidence of nausea, vomiting, and tremors.^42^ The International Parkinson and Movement Disorder Society Evidence‐Based Medicine Committee rated the positive effects of donepezil and galantamine in AD as “potentially useful.”^43^ However, a clinical study inferred that memantine failed to improve cognitive function in patients with PDD.^44^ For AD, memantine was approved by the FDA in 2003 for the treatment of moderate‐to‐severe patients, whereas cholinesterase inhibitors are mainly used for mild‐to‐moderate conditions. In general, a limited number of drugs are available for the treatment of cognitive impairment, and the degree to which these drugs are indicated for cognitive impairment due to different diseases is inconsistent. Therefore, suitable and safe pharmacological choices for treating cognitive impairment are still lacking. Based on this meta‐analysis, we infer that NBP might be used to fill this gap.

What is the underlying mechanism of this phenomenon? Numerous studies have explored the role of NBP. First, the underlying pathological mechanisms of vascular cognitive impairment, the second most common form of cognitive impairment worldwide,^45^ has been identified as oxidative stress, neuroinflammation, and inadequate perfusion of brain tissue.^46^ Chen et al. proposed that NBP could target these factors by reducing the production of reactive oxygen species in hippocampal tissue, inhibiting neuronal apoptosis by regulating the PI3K/AKT signaling pathway, and elevating the expression of Bcl‐2.^47^ Moreover, executive dysfunction can be alleviated in patients with cerebrovascular disease, which might be related to the restoration of chronic hypoperfusion in cerebral white matter after NBP treatment.^48^, ^49^ Second, considering the limited options available for PDD treatment,^50^ several clinical studies have shown that NBP can improve PDD.^32^, ^34^ In addition, NBP can protect dopaminergic neurons by reducing oxidative stress in the PD mouse models,^23^ improving cognitive impairment owing to the involvement of dopaminergic modulation in PD‐related cognitive impairment.^51^ Third, AD is the most predominant disease leading to dementia,^52^, ^53^ and NBP could be a therapeutic approach to modulate oxidative stress and inflammation in APP/PS1 transgenic mice via the Nrf2‐TXNIP‐TrX signaling pathway. Based on the multitarget therapeutic role of NBP, it is essential to believe that NBP has a disease‐modifying effect on cognitive impairment induced by multiple causes.

This meta‐analysis had certain limitations. First, there was some bias. Concerning data assessment, it was difficult to obtain all the raw data for each study. In addition, there might be a selection bias because the studies included were in English, and relevant studies published in other languages were not included. Owing to the limited number of included studies, we did not perform a test for publication bias. Second, there was heterogeneity. Subgroup analysis revealed that the disease type might be a source of heterogeneity; however, other sources of heterogeneity might not have been analyzed, including different basic characteristics of patients, such as sex, age, interventions, duration of therapy, baseline scores of cognitive function, and different diagnostic methods. Third, the inclusion of diseases that cause cognitive impairment was relatively limited. The included studies mainly covered vascular cognitive impairment and PDD, with a lack of other potential causes, such as AD, which might have influenced our findings. Fourth, concerning improvements in the activities of daily living (ADL) scale scores, the inconsistent versions of the ADL used in the included studies rendered it impractical to merge the data and analyze them, thus making it impossible to determine whether NBP had an improving effect on patients' activities of daily living. Finally, evidence for the use of NBP for cognitive impairment is relatively weak. Additional clinical trials are recommended to confirm our findings.

## 5 CONCLUSION

To some extent, NBP (a national class I innovative drug in China) is effective and safe for improving cognitive impairment in patients. However, due to the few relevant clinical studies and their small sample sizes, more large‐sample, multicenter, randomized, and double‐blind studies are needed. In addition, international uniform scales for cognitive impairment assessment should be adopted. Simultaneously, to provide more reliable evidence‐based medicine for cognitive impairment, additional studies are recommended to assess the effects of NBP on specific aspects of cognitive function, such as memory, language, visual space, execution, calculation, and comprehension judgment.

## AUTHOR CONTRIBUTIONS

Qiulu Zhou, Chao Han, and Tao Wang proposed this review and determined the inclusion and exclusion criteria. Yun Xia and Fang Wan searched and collected the studies. Qiulu Zhou and Chao Han extracted data, performed quality assessment, and analyzed the data. Sijia Yin, Yunna Li, Liang Kou, Xiaosa Chi, Junjie Hu, Yadi Sun, Jiawei Wu, Wenkai Zou, and Jinsha Huang drew figures and summarized tables. Qiulu Zhou wrote the first manuscript. Chao Han and Tao Wang further revised and polished the article. All authors contributed to this article and agreed to the final manuscript.

## Funding information

This work was supported by grants 2017YFC1310200 and 2016YFC1306000 from the National Key R&D Program of China (to Tao Wang), grants 81974201 and 81671260 from the National Natural Science Foundation of China (to Tao Wang), and Research supports from Chinese Sleep Research Society Hansoh Project (2019HSA02) (to Jinsha Huang).

## CONFLICT OF INTEREST

The authors declare that the research was conducted in the absence of any commercial or financial relationships that could be construed as a potential conflict of interest.

## Supporting information


Data S1

Table S1

## Data Availability

All data generated or analyzed during this study are included in this published article and its supplementary information files.
