# Supplementary material for: Efficacy and safety of 3‐n‐butylphthalide for the treatment of cognitive impairment: A systematic review and meta‐analysis
Source: CNS Neurosci Ther. 2022 Sep 1;28(11):1706–17. doi: 10.1111/cns.13952 (PMC9532910; doi:10.1111/cns.13952)
Supplement: Supplementary file 2 — Table S1 [file CNS-28-1706-s001.docx]

#1 Cognitive Dysfunction [Mesh]

#2 Cognitive Dysfunctions OR Cognitive Impairments OR Cognitive Impairment OR Mild Cognitive Impairment OR Mild Cognitive Impairments OR Mild Neurocognitive Disorder OR Mild Neurocognitive Disorders OR Cognitive Decline OR Cognitive Declines OR Mental Deterioration OR Mental Deteriorations

#3 #1 OR #2

#4 3-n-butylphthalide [Mesh]

#5 l-NBP cpd OR N-butylphthalide OR butylphthalide OR (S)-(-)-3-butylphthalide OR NBP

#6 #4 OR #5

#7 #3 AND #6
